# Artifact suppression of SSFP cine sequences at 3T using a novel automatic 3D shimming algorithm

**DOI:** 10.1186/1532-429X-15-S1-P57

**Published:** 2013-01-30

**Authors:** Tamara Rothstein, Gabriel C Camargo, Daniel C Quintella, Elsa Fernandes, Ralph Strecker, Andreas Greiser, Maria Eduarda Derenne, Marceu Lima, Joao J Moojen, Patricia B Rizzi, Ronaldo SL Lima, Ilan Gottlieb

**Affiliations:** 1 CDPI - Clínica de Diagnóstico por Imagem, Rio de Janeiro, Brazil; 2 Siemens Ltda, São Paulo, Brazil; 3 Siemens Healthcare, Erlangen, Germany

## Background

Imaging the heart at 3T has some advantages, but magnetic susceptibility increases at higher field strengths, generating deleterious banding artifacts, especially when using SSFP-based sequence. We aimed to evaluate whether a recently developed advanced shimming algorithm reduces banding artifacts on SSFP cine images at 3T.

## Methods

**Figure 1:**
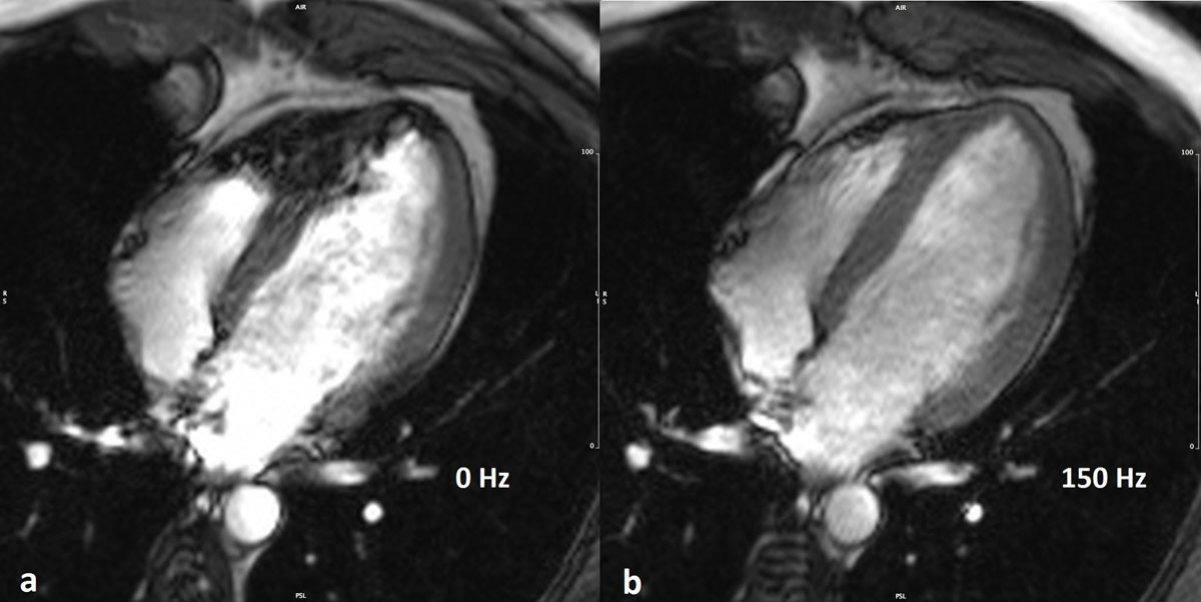
Frequency scout images of the four chamber view. Banding artifacts are seen at frequency offset of 0 Hz (a), but not at 150 Hz (b).

A total of 20 consecutive patients scheduled to undergo a clinically indicated CMR in normal sinus rhythm and able to perform apnea were prospectively enrolled to be scanned in a 3T system (MAGNETOM Verio, Siemens, Germany). Frequency scout images were acquired in two and four chamber views and the frequency offset associated with least banding artifacts involving the heart was manually set (Figure 1) for the acquisition of short and long axis segmented cine SSFP images covering the entire ventricular volumes (bandwidth of 700-750 Hz/pixel and echo spacing of 3.4 ms). The same sequences were then repeated with the offset reset to 0 Hz (only change made) but with the use of a novel 3D shimming algorithm restricted to the heart (instead of the entire FOV) based on a patient specific GRE fieldmap previously generated (WIP - Siemens, Germany).

Images were blindly evaluated for segmental function (normal vs. abnormal), and the presence of artifacts within the heart (none, minor or important - the latter meaning that at least one myocardial segment could not be assessed). Ventricular volumes were also blindly measured according to guidelines. Categorical differences were assessed with chi-square, kappa was calculated for agreement and continuous variables were assessed using Student's T test.

## Results

Mean LV and RV volumes and ejection fraction (EF) were not statistically different between acquisition modes, and had small biases between measurements (Table 1). Segmental wall motion analysis had good agreement with kappa of 0.76, p<0.001. Minor artifacts were seen in at least one segment in 4 (20%) patients using the new shimming and 7 (35%) using frequency offsets. Important artifacts were seen in at least one segment in 3 (15%) patients using the new shimming and 2 (10%) using frequency offsets.

**Table 1:** Mean LV and RV volumes and ejection fraction.

	with shim	without shim	p-value	bias*
LVEDV	139,0	133,5	0,67	5,5
LVESV	60,0	60,1	0,99	-0,1
LVEF	59,5%	59,0%	0,91	0,5%
RVEDV	127,5	124,6	0,77	2,9
RVESV	60,0	57,5	0,70	2,5
RVEF	53,7%	55,0%	0,57	-1,3%

* with shim - without shim

## Conclusions

Cine SSFP at 3T with a new shimming algorithm targeted to the heart is similar to the standard practice of selecting the best frequency offset. This new shimming might simplify cardiac acquisitions at 3T.

## Funding

Internal

